# CProMG: controllable protein-oriented molecule generation with desired binding affinity and drug-like properties

**DOI:** 10.1093/bioinformatics/btad222

**Published:** 2023-06-30

**Authors:** Jia-Ning Li, Guang Yang, Peng-Cheng Zhao, Xue-Xin Wei, Jian-Yu Shi

**Affiliations:** School of Life Sciences, Northwestern Polytechnical University, Xi’an 710072, China; School of Life Sciences, Northwestern Polytechnical University, Xi’an 710072, China; School of Life Sciences, Northwestern Polytechnical University, Xi’an 710072, China; School of Life Sciences, Northwestern Polytechnical University, Xi’an 710072, China; School of Life Sciences, Northwestern Polytechnical University, Xi’an 710072, China

## Abstract

**Motivation:**

Deep learning-based molecule generation becomes a new paradigm of *de novo* molecule design since it enables fast and directional exploration in the vast chemical space. However, it is still an open issue to generate molecules, which bind to specific proteins with high-binding affinities while owning desired drug-like physicochemical properties.

**Results:**

To address these issues, we elaborate a novel framework for controllable protein-oriented molecule generation, named CProMG, which contains a 3D protein embedding module, a dual-view protein encoder, a molecule embedding module, and a novel drug-like molecule decoder. Based on fusing the hierarchical views of proteins, it enhances the representation of protein binding pockets significantly by associating amino acid residues with their comprising atoms. Through jointly embedding molecule sequences, their drug-like properties, and binding affinities w.r.t. proteins, it autoregressively generates novel molecules having specific properties in a controllable manner by measuring the proximity of molecule tokens to protein residues and atoms. The comparison with state-of-the-art deep generative methods demonstrates the superiority of our CProMG. Furthermore, the progressive control of properties demonstrates the effectiveness of CProMG when controlling binding affinity and drug-like properties. After that, the ablation studies reveal how its crucial components contribute to the model respectively, including hierarchical protein views, Laplacian position encoding as well as property control. Last, a case study w.r.t. protein illustrates the novelty of CProMG and the ability to capture crucial interactions between protein pockets and molecules. It’s anticipated that this work can boost *de novo* molecule design.

**Availability and implementation:**

The code and data underlying this article are freely available at https://github.com/lijianing0902/CProMG.

## 1 Introduction

During the drug design, it is essential to screen or design candidate compounds binding to protein targets. However, it is extremely difficult to find appropriate small molecules in the vast chemical space, including 10^23^–10^60^ compounds as estimated (Polishchuk et al. 2013). In past years, high-throughput screening (Macarron et al. 2011) and virtual screening (Schneider and Böhm 2002) are two classical techniques of computer-aided drug design, which search candidate molecules in predefined compound libraries. However, they only perform limited searching in chemical space such that their finding molecules are not novel due to predefined small-size molecule libraries. In recent years, biologists and pharmacologists have been paying attention to various deep generative models, which have been successfully used in computer vision and natural language processing. They believe that the design of novel small molecules via deep generative models (called molecule generation) can explore the entire chemical space. Molecule generation provides a new paradigm of *de novo* molecule design.

Current deep learning-based molecule generation methods can be roughly categorized into ligand-based and protein-based methods.

(1) Ligand-based methods

By learning hidden chemical rules of structure forming among existing small molecules, ligand-based methods generate novel molecules different from them. Typically, ligand-based methods leverage three types of generative models, including Recurrent Neural Networks (RNN) (Graves 2014), Generative Adversarial Networks (GAN) (Creswell et al. 2018), and Variational Autoencoders (VAE) (Kingma and Welling, 2022).

Since RNN naturally processes variable-length sequences, it can generate novel molecules by representing molecules in SMILES strings. Furthermore, aiming to refine generated molecules having desired drug-like properties, RNN-based methods always employ diverse optimization strategies. For example, ChemTS directly applies an RNN to generate novel molecules, which are further optimized by a tree search to find molecules having specific drug-like properties (Yang et al. 2017). Based on the pretraining strategy, RNN can be fine-tuned by transfer learning (Segler et al. 2018) or reinforcement learning (Wang et al. 2021) to generate property-specific molecules. In contrast to these optimization strategies, the training of a conditional RNN by setting its initial state with specific molecular properties can directly generate novel property-specific molecules (Kotsias et al. 2020). However, RNN is designed for sequences but not for graphs (e.g. molecule structures).

GAN and VAE are two typical distribution-based generative models, which can characterize the small molecule space. GAN contains a generator and a discriminator, contesting with each other by a zero-sum (adversarial) game. They are trained together in an adversarial manner which enables the generation of novel molecules. ORGAN (Guimaraes et al. 2018) utilizes a SMILES-based GAN to generate molecules. The pioneering graph-based model, MolGAN (De Cao and Kipf 2018), employs a GAN to directly generate molecular graphs. A reinforcement learning module is a popular strategy to help generate molecules with specific properties. However, the training of GAN usually suffers from mode collapse.

VAEs are generative encoder–decoder models under explicit normal distribution assumptions. Since the latent distribution space is analogous to the chemical space, the designated sampling in it enables the generation of novel molecules owning specific properties. There are various approaches to generate novel molecules with desired properties, such as an extra property predictor (Gómez-Bombarelli et al. 2018) and a conditional VAE (Lim et al., 2018). Considering molecule structures contain richer information than SMILES strings, some works directly generate novel molecular structures but not SMILES strings. For example, GraphVAE (Simonovsky and Komodakis 2018) design a graph-based VAE by representing molecules as graphs with attributes. JT-VAE (Jin et al. 2018) combine a tree-structured scaffold over chemical substructures into a molecule with a graph message-passing network. Remarkably, VAE-generated molecules only exhibit moderate novelty and diversity.

Ligand-based methods can generate novel compounds with favorable physicochemical properties. However, since they consider no or less protein information when generating molecules, they cannot guarantee that generated molecules have desired binding affinity to new protein targets.

(2) Protein-based methods

In contrast, recent protein-based methods ensure that generated molecules bind to specific protein targets with high-binding affinities. Some methods turn such a generation into a machine translation problem, which translates protein sequences (amino acid sequences) into molecule sequences (SMILE strings). In this context, Transformer can be directly applied for protein-based molecule generation (Grechishnikova 2021). AlphaDrug (Qian et al. 2022) improves the vanilla Transformer by multiple skip connections from its protein encoder to its molecule decoder to obtain better protein representations and further applies the tree search to guide molecule generations. However, only considering protein sequences, these methods neglect the information in binding pockets, which imply how a molecule binds to a protein.

Some methods attempt to utilize protein 3D structures when generating molecules. For example, based on the voxelization of 3D protein pockets and 3D molecule structures, Skalic et al. (2019) trains a GAN to generate 3D shapes of molecules, which are further decoded into multiple candidate SMILES strings by a captioning network. Recently, Xu et al. (2021) construct a protein residue-based Coulomb matrix to directly characterize the spatial structure of the pocket, which is further input into a conditional RNN to control the generation of molecules.

To enhance the protein structure representation, recent works characterize the binding interface between a protein and a molecule. By considering the 3D coordinates of atoms in given binding sites, Luo et al. (2021) design a 3D generative model to estimate the probability density of atom occurrences in the 3D binding space, and perform an autoregressive sampling scheme on the binding spatial locations assigned with higher probabilities to generate molecules atom by atom. But this approach ignores bond types and functional groups in the binding pocket. To solve the problem, its extension, Pocket2Mol (Peng et al. 2022) designed an E(3) equivariant neural network to capture spatial and bonding relationships between atoms in the binding pocket.

However, the representation of 3D structures is still challenging so far. In terms of binding affinity, the molecules they generated are surprisingly lower than those generated by 1D sequences (Qian et al. 2022). More importantly, it is difficult to generate small molecules w.r.t. drug-like physicochemical properties under control.

To address the above issues, we elaborate a protein-oriented generative framework (CProMG), which contains a 3D protein embedding module, a dual-view protein encoder, a molecule embedding module, and a novel drug-like molecule decoder. Overall, the main contributions of our CProMG are as follows.

1. It serves in a controllable learning framework to generate novel small molecules having high-binding affinities to specific protein targets while owning desired drug-like properties.
2. It provides a better representation of 3D protein structure (pocket) by integrating a fine-grained atom view with a coarse-grained amino acid view based on an interactive attention block in the encoder.
3. It leverages the protein-interactive multi-head attention block in the decoder to calculate the proximity of molecule tokens to protein residues and atoms, such that crucial interactions between protein pockets and molecules can be captured.

## 2 Materials and methods

### 2.1 Problem formulation and model construction

Suppose that *m* proteins $P=\{p_{i},i=1,\;2,\ldots ,m\}$ bind to *n* small molecules $C=\{c_{j},j=1,\;2,\ldots n\}$. Let $a_{i,j}$ be the binding affinity of $p_{i}$ with respect to $c_{j}$. In addition, $c_{j}$ has specific physicochemical properties $y_{j}\in R^{{1\times d}_{y}},\;y_{j}(t)\in \{1,0\}\;\text{or}\;y_{j}(t)\in R,j\in \{1,\;2,\ldots n\}$. The former type of $y_{i}(j)$ indicates a hard (binary/discrete) property of $c_{j}$ (e.g. Synthetic Accessibility, SA), while the latter represents its soft (continuous) properties (e.g. logP). For example, a molecule entry, named PF-4989216, assigned with the compound ID 51033720 in PubChem, has a value of LogP = 2.919. Meanwhile, it has a good SA score (i.e. 0.78). In practice, since pharmacologists are more interested in whether the molecule can be synthesized easily, SA is binarized by the rule that SA = 1 if SA≤4.0 (i.e. easy to be synthesized), otherwise SA = 0 (i.e. difficult to be synthesized) (Wang et al. 2021). We consider two types of molecule properties simultaneously when generating novel molecules.

**Figure 1.**
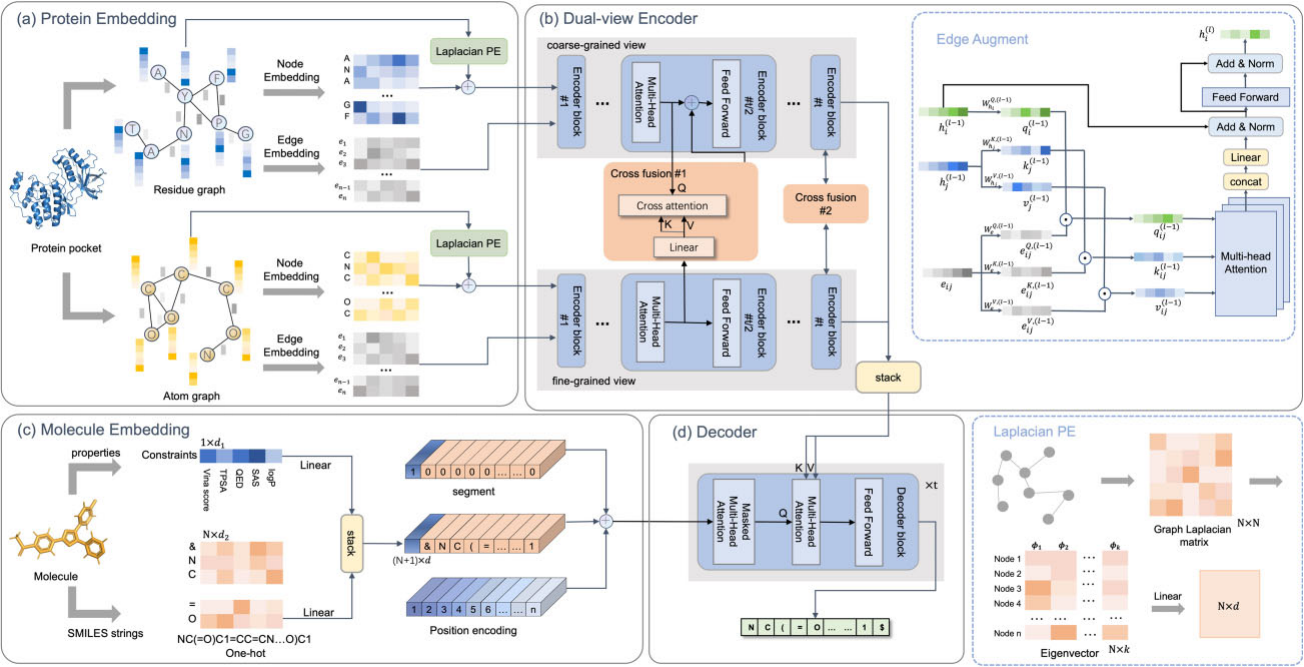
The framework of CProMG. This framework is composed of four modules, a 3D protein graph embedding module, a dual-view protein encoder, a drug-like molecule embedding module, and a novel molecule decoder. (a) Protein embedding module. A protein (pocket) is represented in a residue graph and an atom graph in parallel. Nodes and edges in each protein graph are embedded. Especially, nodes have additional Laplacian positional encodings. Node representations are also augmented by edge representations. (b) Dual-view protein encoder. It contains two parallel encoder modules w.r.t. protein graph, of which each module is composed of *t* encoding blocks. Each block contains a multi-head self-attention unit and a feedforward neural network. There are also two cross-attention units between the parallel encoder modules. The concatenation of representations of two encoder modules is output as the protein representation and input into the molecule decoder as the key and value. (c) Molecule embedding module. It encodes physicochemical properties of small molecules, docking scores w.r.t. proteins, and their SMILES sequences simultaneously. The concatenation of them is added with an extra positional encoding as the Query input into the decoder. (d) Molecule decoder. It contains *t* decoder blocks, each of which contains a masked multi-head attention unit, a cross-attention unit, and a feed-forward network. The decoder autoregressively predicts the next token of the molecular sequence through the generated molecular intermediates and proteins representation.

Given a new protein $p_{x}$, the task is to generate a set of novel molecules $\left\{c_{x}^{k},k=1,\;2,\ldots \right\}$, which bind to $p_{x}$ with high-binding affinities and have desired physicochemical properties $y_{x}$. Inspired by Transformer (Vaswani et al. 2017), we treat this task as a specific translation from proteins into small molecules. We design a novel protein-oriented molecule generation framework, including a 3D protein graph embedding module, a dual-view protein encoder, a drug-like molecule embedding module, and a novel molecule decoder (Fig. 1).

### 2.2 Protein graph embedding module

Inspired by the hierarchy of protein structure (Jin et al. 2022; Wang et al. 2023), we characterize 3D protein structures in both an amino acid view and an atom view. To reduce the computation, only binding pockets are considered when characterizing 3D protein structures.

(a) Protein graph construction

Technically, given a protein p, its 3D structure can be represented as a graph G=(V,E). Specifically, $V={\left\{(v_{i},r_{i})\right\}}_{i=1}^{n}$ is the node set, where a node $v_{i}$ has known 3D coordinates $r_{i}\in R^{3}$ and n denotes the number of nodes. Moreover, $E=\{e_{ij},i,j=1,\;2,\ldots ,n\;\&\;i\neq j\}$ denotes a set of edges between nodes. We build an amino acid residue-based graph ($G_{r}$) and an atom-based graph ($G_{a}$), respectively.

In the residue-based graph $G_{r}$, we treat amino acid residues as the nodes V. Each node $v_{i}$ is naturally represented as a one-hot coding vector $x_{i}$ according to 20 amino acid types. The edges between them are determined by their Euclidean distances. In detail, being the representative point of $v_{i}$, its centroid is first calculated by $c_{i}=\sum \left(r_{i}^{k}*m_{i}^{k}\right)/\sum m_{i}^{k},k=1,\;2\ldots ,\;$where $\left\{r_{i}^{k}\right\}$ represent the atom coordinates in $v_{i}$ and $\left\{m_{i}^{k}\right\}$ are its atomic masses accordingly. Then, the pairwise Euclidean distance between $v_{i}$ and $v_{j}$ is calculated by $d_{i,j}={\left\|c_{i}-c_{j}\right\|}_{2}$. It is used further to construct edges $\left\{e_{ij},j\in N_{i}\right\}$ by the K-nearest neighbor (KNN) algorithm, which selects *k* nearest neighbor nodes $\left\{v_{j}\in N_{i}\right\}$ (e.g. *k *=* *48), where $N_{i}$ is the node neighborhood of $v_{i}$. Last, $d_{i,j}$ is set as the initial representation of $e_{ij}$. The residue view of a protein provides a coarse-grained representation of its binding pocket.

In the atom-based graph $G_{a}$, we treat atoms as the nodes V. Similarly, each atom is represented by the one-hot encoding based on six popular atom types, including H, C, N, O, S, and P. Moreover, each atom in the protein backbone is annotated by an additional bit, where 1 indicates its location being in the backbone, and 0 otherwise. Thus, each node $v_{i}$ is represented as a 7-dimensional vector ($x_{i}$). We determine an edge between two nodes in a similar way as that in $G_{r}$ but with a different number of nearest neighbors (i.e. *K* = 30) as recommended by Ingraham et al. (2019). The atom view provides a fine-grained representation of its binding pocket.

Once the protein graphs are built, our task is to generate the embeddings of nodes and edges. In common, suppose that each node $v_{i}$ has the initial representation $x_{i}\in R^{1\times d_{v}}$ and each edge $e_{ij}$ has the initial representation $d_{ij}\in R^{1\times 1}$. The embedding of $v_{i}$ (i.e. $h_{i}^{\left(0\right)}\in R^{1\times d}$) is defined as where $h_{i}^{\mathrm{pos}}\in R^{1\times d}$ is the Laplacian positional encoding vector of $v_{i}$, and $W^{\left(0\right)}\in R^{d_{v}\times d}$ is the learnable parameter. See Section 2.2(b) for the definition of $h_{i}^{\mathrm{pos}}$. Inspired by the idea analogous to RBF neural network (Seshagiri and Khalil 2000), we obtain the embeddings of $e_{ij}$ by $d_{e}$ RBFs mapping its initial representation $d_{i,j}$ into $e_{ij}\in R^{1\times d_{e}}$.


(1) $$h_{i}^{\left(0\right)}=x_{i}W^{\left(0\right)}+h_{i}^{\mathrm{pos}},$$

When we attempt to encode each node in a protein graph to obtain a unique positional representation of each node, however, it’s hard to directly define the positions of nodes in a graph. In other words, we cannot apply the positional coding in the vanilla Transformer to the protein graphs. To cope with this issue, we borrow the idea of the Laplacian position encoding in graph neural networks to obtain unique positional representations of nodes in the following.

(b) Laplacian positional encoding

Because any signal can be represented as a combination of sine/cosine functions with varying frequencies, Transformer regards the positional coding as a Fourier Transform on a signal. As a result, each entity in turn is coded into a position-unique vector. However, such positional coding cannot be directly applied to graphs because it’s hard to define the positions of nodes in a graph. To cope with this issue, we leverage the Laplacian position encoding in graph neural networks (Kreuzer et al. 2021) to assign each node in a graph with a unique representation.

Given a weighted graph G=(V,E,W), the weight $w_{ij}$ of each edge $e_{ij}$ is defined as $w_{ij}=e^{-d_{ij}^{2}/\left(2\sigma^{2}\right)}$, where the hyperparameter σ is empirically set as 30 in $G_{r}$ and 15 in $G_{a}$ respectively. Its Laplacian matrix $L\in R^{n\times n}$ can be defined as follows (Dwivedi et al. 2022): where $I\in R^{n\times n}$ is the identity matrix, the n×n diagonal matrix D represents the degree matrix of weighted graph G, its *k*-th element $d_{k,k}$ is the degree of the *k*-th node and A represents the weighted adjacency matrix of G, the n×n diagonal eigenvalue matrix Λ contains eigenvalues$\left\{\lambda_{k}\right\}$ from small to big along with its diagonal, $U\in R^{n\times n}$ contains a set of eigenvectors $\left\{u_{k}\in R^{n\times 1}\right\}$ w.r.t. $\left\{\lambda_{k}\right\}$ and $u_{k}$ is normalized to unit length (i.e. $u_{k}^{T}u_{k}=1$). Thus, L enables the Fourier Transform on the graph G. Specifically, the eigenvectors $\left\{u_{k}\right\}$ of L, analogous to sine/cosine functions (Dwivedi and Bresson 2021), can be regarded as the basis vectors to encode the positions of nodes in G. The eigenvalue is considered as a node position in the Fourier domain of the graph (Bronstein et al. 2017).


(2) $$L=I-D^{-\frac{1}{2}}AD^{-\frac{1}{2}}=U^{T}\Lambda U,$$

In the spectral graph theory, eigenvalues can be used to discriminate between different graph structures and substructures, as they can be interpreted as the frequencies of resonance of the graph (analogous to the frequencies reflected by sine/cosine functions again) (Kreuzer et al. 2021). Accordingly, smaller eigenvalues (frequencies) are more heavily weighted when determining distances between nodes. Moreover, corresponding low-frequency eigenvectors are spread across the graph, while higher frequencies often resonate in local structures (Kreuzer et al. 2021). Therefore, we take low-frequency eigenvectors of nodes w.r.t. the first k-smallest eigenvalues [e.g. *k *=* *8 (Kreuzer et al. 2021)] as their positional features.

For each node $v_{i}$, its positional encoding $h_{i}^{\mathrm{pos}}$ is defined as: where U(i,1:k) is the positional vector consisting of the first *k* elements in the *i*-th row of U, and the learnable $W^{\mathrm{pos}}\in R^{k\times d}$ works like an adapter to map the positional coding from the eigenspace to the node embedding space. Such a coding can capture the intuition that nodes far apart are different whereas nodes nearby are similar in terms of positional features.


(3) $$h_{i}^{\mathrm{pos}}=U(i,1:k)W^{\mathrm{pos}},$$

### 2.3 Dual-view encoder

The embeddings of the amino acid graph $G_{r}$ and that of the atom graph $G_{a}$ are input separately into the dual-view encoder to obtain the final representation of the protein binding pocket. The dual-view encoder contains two parallel encoders ${\mathrm{En}}_{r}$ and ${\mathrm{En}}_{a}$ accounting for the encodings of two graphs $G_{r}$ and $G_{a}$, respectively. Each encoder is composed of *t* tandem encoding units, of which each contains an edge-augmented encoding block $E_{a}^{t}$ and a multi-head attention block $M_{a}^{t}$. The first block $E_{a}^{t}$ enhances node representations while the attention block $M_{a}^{t}$ further updates node representations by a self-attention mechanism.

Remarkably, $G_{r}$ and $G_{a}$ represent the coarse-grained (residues) and the fine-grained (atoms) information of the protein binding pocket respectively. As a result, the dual-view encoder also leverages two cross-attention blocks between ${\mathrm{En}}_{r}$ and ${\mathrm{En}}_{a}$ to fuse the coarse-grained representation of the protein binding pocket with its fine-grained representation. Such a fusion helps capture the natural protein structure hierarchy.

(1) Graph encoders

Technically, suppose that node $v_{i}$ and its neighboring nodes $\left\{v_{j}\right\}$, where $j\in N_{i}$ and $N_{i}$ is the neighborhood of $v_{i}$. For the l-th encoding unit, the edge-augmented encoding block $E_{a}^{l}$ enhances their representations to accommodate the self-attention framework by two steps. The first step maps the node representation $h_{i}^{\left(l-1\right)}$ output by the previous encoding unit into the Query $q_{i}^{\left(l\right)}$ and maps $h_{j}^{\left(l\right)}$ into the Key $k_{j}^{\left(l\right)}$ and the Value $v_{j}^{\left(l\right)}$ in parallel. The second enhances $q_{i}^{\left(l\right)}$, $k_{j}^{\left(l\right)}$, and $v_{j}^{\left(l\right)}$ by the edge representation $e_{ij}$. For each neighboring node $v_{j}$ of $v_{i}$, we define neighbor-specific Queries $q_{ij}^{\left(l\right)}\in R^{1\times d_{k}}$, Keys $k_{ij}^{\left(l\right)}\in R^{1\times d_{k}}$, and Values $v_{ij}^{\left(l\right)}\in R^{1\times d_{v}}$ as: where $q_{i}^{\left(l\right)}=h_{i}^{\left(l-1\right)}W_{h}^{Q(l)}$, $k_{i}^{\left(l\right)}=h_{i}^{\left(l-1\right)}W_{h}^{K(l)}$, $v_{i}^{\left(l\right)}=h_{i}^{\left(l-1\right)}W_{h}^{V(l)}$ and $\left\{W\right\}$ are learnable matrices accounting for specific linear transformations, and ⊙ denotes the element-wise multiplication. Specifically, $h_{i}^{\left(0\right)}$ is the node representation output by the protein graph embedding module and input into the first encoding unit. See also the Edge Augment in Fig. 1.


(4) $$q_{ij}^{\left(l\right)}=q_{i}^{\left(l\right)}⊙\left(e_{ij}W_{e}^{Q\left(l\right)}\right),\;k_{ij}^{\left(l\right)}=k_{i}^{\left(l\right)}⊙\left(e_{ij}W_{e}^{K\left(l\right)}\right),\;v_{ij}^{\left(l\right)}=v_{i}^{\left(l\right)}⊙\left(e_{ij}W_{e}^{V(l)}\right),$$

Suppose that multi-head attention block $M_{a}^{l}$ in the l-th encoding unit contains H heads of parallel attention layers. It updates the node representation as $h_{i}^{\left(l\right)}={\left|\right|}_{r=1}^{H}\left(h_{i,r}^{\left(l\right)}\right)$, where $h_{i,r}^{\left(l\right)}\;$accounts for the output of its *r*-th attention layer and defined as


(5) $$h_{i,r}^{\left(l\right)}=\sum_{j\in N_{i}}\left({\mathrm{softmax}}_{j}(\frac{q_{ij}^{\left(l\right)}{\left(k_{ij}^{\left(l\right)}\right)}^{T}}{\sqrt{d_{k}}})v_{ij}^{\left(l\right)}\right).$$

Then, inspired by the encoder in the Transformer, the updated node representation $h_{i}^{\left(l\right)}$ is also mapped by a linear layer and combined with $h_{i}^{\left(l-1\right)}$ as a residual connection (i.e. $h_{i}^{\left(l\right)}\leftarrow \eta \left(h_{i}^{\left(l\right)}W_{h}^{\left(l\right)}+h_{i}^{\left(l-1\right)}\right)$), where η(·) represents the normalization function (i.e. LayerNorm). After that, a feed-forward network (FNN) with a residual connection is used to update it further (i.e. $h_{i}^{\left(l\right)}\leftarrow \eta \left(\mathrm{FNN}(h_{i}^{\left(l\right)})+h_{i}^{\left(l\right)}\right)$). Furthermore, the updated node representation matrix $H_{}^{\left(l\right)}$ stacked by $\left\{h_{i}^{\left(l\right)}\right\}$ is output and used as the input of the next encoding unit if l<t. Last, supposing that the node representation matrices derived from two encoders ${\mathrm{En}}_{r}$ and ${\mathrm{En}}_{a}$ are $H^{\left(t\right)}$ and $Z^{\left(t\right)},$ respectively, we vertically stack them as the final representation matrix $H_{P}$ of the protein structure (i.e. $H_{P}=\left[H^{\left(t\right)};Z^{\left(t\right)}\right]$).

(2) Dual-view fusion

During the parallel encoding process, we designed a one-way cross-fusion block between the l-th encoding unit in ${\mathrm{En}}_{r}$ and that in ${\mathrm{En}}_{a}$, which updates coarse-grained $H^{\left(l\right)}$ by fine-grained $Z^{\left(l\right)}$. Specifically, it treats $\left\{z_{j}^{\left(l\right)}\right\}$ as both the Keys and the Values while treating $\left\{h_{i}^{\left(l\right)}\right\}$ as the Queries. Such a fusion block provides an extra advantage that an amino acid residue can be associated with its comprising atoms.

The fusion block is also a multi-head attention block. Let $h_{i}^{\left(l\right)}$ be the amino acid representation output by the l-th attention block in ${\mathrm{En}}_{r}$, $z_{j}^{\left(l\right)}$ be the atom representation output by the l-th attention block in ${\mathrm{En}}_{a}$, $H_{f}$ be the number of heads in the fusion block. The Query $q_{i}^{\left(l\right)}\in R^{1\times d_{k}}$, the Key $k_{j}^{\left(l\right)}\in R^{1\times d_{k}}$ and the Value $v_{j}^{\left(l\right)}\in R^{1\times d_{v}}$ w.r.t. l are defined as where three *W* matrices represent linear layers. Thus, the fusion block updates amino acid representations by the concatenation ${\left|\right|}_{r=1}^{H_{f}}\left(h_{i,r}^{\left(l\right)}\right)$, where $h_{i,r}^{\left(l\right)}\;$accounts for the output of its *r*-th attention layer and is defined as


(6) $$q_{i}^{\left(l\right)}=h_{i}^{\left(l\right)}W_{f}^{Q(l)},\;k_{j}^{\left(l\right)}=z_{j}^{\left(l\right)}W_{f}^{K(l)},\;v_{j}^{\left(l\right)}=z_{j}^{\left(l\right)}W_{f}^{V(l)},$$

(7) $$h_{i,r}^{\left(l\right)}=\sum_{j=1}^{n}\left({\mathrm{softmax}}_{j}(\frac{q_{i}^{\left(l\right)}{\left(k_{j}^{\left(l\right)}\right)}^{T}}{\sqrt{d_{k}}})v_{ij}^{\left(l\right)}\right).$$

Similarly, the concatenation is mapped further by another linear layer as ${\hat{h}}_{i}^{\left(l\right)}=\left({\left|\right|}_{r=1}^{H_{f}}\left(h_{i,r}^{\left(l\right)}\right)\right)W_{f}^{\left(l\right)}$, and further works as a residual connection to update the original $h_{i}^{\left(l\right)}$ by $h_{i}^{\left(l\right)}\leftarrow \eta \left(h_{i}^{\left(l\right)}+{\hat{h}}_{i}^{\left(l\right)}\right)$, where η(·) is the normalization function.

To reduce the information redundancy, we only build two cross-fusion blocks for a middle encoding unit (e.g. the 3rd unit) and the last encoding unit (e.g. the 6th unit), respectively.

### 2.4 Molecule embedding module

The molecule embedding module encodes molecule sequences based on a pre-built vocabulary and encodes their drug-like properties as well as binding affinities w.r.t. proteins simultaneously. The molecule decoder can generate novel molecules owning desired properties in a controllable manner.

We utilize the tokenization proposed by Schwaller et al. (2018) to build a vocabulary V, which contains k non-overlapping “words” (substrings in the SMILE string, or called tokens), such that each SMILES string is turned into a sequence of words.

Formally, given the *n*-length SMILES sequence of a small molecule c, it can be turned into an n-word sequence $s_{c}=\{a_{1},.,a_{n}\}$, where $a_{i}\in V$. To perform the decoding, we add a prefix tag on this word sequence as $s_{c}^{*}=\{{b,a}_{1},.,a_{n}\}$, where b∈V is the beginning tag when starting the decoding. Accordingly, $s_{c}^{*}$ is represented as an (n+1)×k one-hot encoding matrix S based on the vocabulary V.

Let $\left\{p_{1},.,p_{m}\right\}$ be the property sequence of the molecule c, where each character indicates one of its property names (e.g. Synthetic Accessibility, LogP,…), and $y\in R^{1\times m}$ be the vector of property values, where y(t)∈{1,0} or y(t)∈R. The former type of y(t) indicates hard properties (e.g. Synthetic Accessibility), while the latter represents soft properties (e.g. logP). To generate novel molecules with better docking in proteins of interest, we binarize the docking scores S of protein–ligand pairs as a hard property. Specifically, S=1 if S≤-7.5, and 0 otherwise. Thus, we obtain the molecule representation $h^{m}\in R^{(n+2)\times d}$ by where “;” is a stacking operation of matrices, $W_{p}\in R^{m\times d}$ and $W_{s}\in R^{k\times d}$ account for two linear layers, respectively.


(8) $$h^{m}=[yW_{p};SW_{s}],$$

Moreover, we add a property tag “p” at the head of $s_{c}^{*}$ to indicate the molecule with properties as $s_{p}=\{p,b,a_{1},.,a_{n}\}$. This is a crucial trick to make the generation of novel molecules owning desired properties. See also Section 2.5 for detailed reasons. To describe such a sequence briefly, we regard a tag or a word in it as a token, which is assigned with a binary type indicator (i.e. 1 for property and 0 for word). Accordingly, two token types are also embedded as vectors $t_{1},t_{0}\in R^{1\times d}$. The token type representation of the molecule is defined as the stacking of token type embedding w.r.t. $s_{p}^{*}$,


(9) $$h^{\mathrm{token}}=\left[t_{1};t_{0};\ldots ;t_{0}\right].$$

Furthermore, we consider the positional relationship among tokens. Inspired by the Transformer (Vaswani et al. 2017), we use sine and cosine functions of different frequencies to encode the position of the i-th token into a d-dimensional unique representation $h_{i}^{\mathrm{pos}}\in R^{1\times d}$ as follows: where j=1, 2,…,N, N=d/2 if *d* is an even number or $N=\left(d+1\right)/2$ if an odd number. The wavelengths form a geometric progression from 2π to *r*·2π, where *r* = 10 000 as suggested by Vaswani et al. (2017). Thus, the positional representation of the molecule $H_{}^{\mathrm{pos}}$ is just the stack of $\left\{h_{i}^{\mathrm{pos}}\right\}$.


(10) $$h_{i}^{\mathrm{pos}}\left(2\left(j-1\right)+1\right)=\text{cos}(\frac{i}{r^{2j/d}}),\;h_{i}^{\mathrm{pos}}\left(2j\right)=\text{sin}(\frac{i}{r^{2j/d}}),$$

Finally, the whole embedding of small molecule *c* is defined as:


(11) $$h^{0}=h^{m}+h^{\mathrm{token}}+H^{\mathrm{pos}}.$$

### 2.5 Decoder and molecule generation

We directly adopt the same architecture of the decoder as that of the original Transformer, which contains *t* tandem decoding units. Each unit is composed of a masked multi-head attention block, a protein-interactive multi-head attention block, and an ordinary multilayer perceptron. The masked module prevents the decoder from information leakage when predicting the next token. The interactive module calculates the proximity of molecule tokens to protein residues and atoms by regarding the former as queries and the latter as keys and values in an attention layer. See also Vaswani et al. (2017) for details.

The molecule generation is completed by an autoregressive decoding process, which begins with the sequence of two tokens {p,b,*,…,*} in and iteratively appends potential tokens $a_{i}^{*}$ to it one by one until the ending tag “e” (i.e. $\left\{p,b,a_{1}^{*},.,a_{t}^{*},e\right\}$). The resulting sequence $\left\{a_{1}^{*},.,a_{t}^{*}\right\}$ is directly taken as the SMILES string of the novel molecule.

As remarked in Shuai et al. (2021) and Madani et al. (2021), the molecular property token should be put in the head of the token string because the essence of the autoregressive decoding is an iterative process under progressive conditional probabilities where properties are the first condition to generate the next molecular token. Such a crucial step guarantees the molecular generation controllable w.r.t. properties.

The training of the model aims to maximize the following negative log-likelihood: where $x_{0}=[p,\;b]$, and $x_{i}$ is the token in $s_{p}$. In the generation, the model generates novel molecules based on the learned conditional probability distribution as where $x_{0}=[p,b],\;x_{m}^{*}=\prime \;e\prime ,$$x_{i}^{*}$ is the generated token. Finally, complete generated token strings $\left\{s_{p}^{*}\right\}$ are obtained by top-k high conditional probabilities, and their substrings $\left\{x_{1}^{*},x_{2}^{*},\ldots ,x_{i-1}^{*}\right\}$ are corresponding SMILES strings, such that protein-oriented novel molecules (with high-binding affinities and desired properties) are generated.


(12) $$L\left(D\right)=-\sum_{i=1}^{n}\;\log P\left(x_{i}|\left[x_{0},x_{1},x_{2},\ldots ,x_{i-1}\right],G_{r},G_{a}\;\right),$$

(13) $$P(s_{p}^{*})=\prod_{i=1}^{m}\;P\left(x_{i}^{*}|\left[x_{0},x_{1}^{*},x_{2}^{*},\ldots ,x_{i-1}^{*}\right],G_{r},G_{a}\right),$$

### 2.6 Evaluation metrics

To evaluate the performance of molecule generation models, we follow the conventional settings in recent works (Bagal et al. 2021; Luo et al. 2021), which use Vina Score (VS), High Affinity Ratio (HAR), Quantitative Estimate of Drug-likeness (QED), Synthetic Accessibility Score (SA), Diversity, Water-Octanol Partition Coefficient (logP), Molecular Weight (MW) as the performance metrics. They are introduced as follows.

VS measures the average binding affinity between generated molecules and proteins of interest. We use Autodock Vina (Trott and Olson 2010) to calculate docking scores. Since the docking score is negative, the less, the better.

SA reflects the average difficulty of synthesizing a given molecule by its synthesizable fragments (Ertl and Schuffenhauer 2009). A drug-like molecule usually has SA≤4.0. The lower, the easier to be synthesized.

HAR indicates the percentage of generated molecules having higher binding scores than those of reference molecules or equal to them. The greater, the better.

QED measures the average similarity between generated molecules and existing drugs by multiple chemical attributes (Bickerton et al. 2012). Its value falls into $\left[0,1\right]$. The greater, the better.

Diversity evaluates the diversity within a group of generated molecules G in terms of chemical structure, and its definition is as $\mathrm{Diversity}=1-\frac{1}{N^{2}}\sum_{m_{1},m_{2}\in G}\;T\left(m_{1},m_{2}\right)$, where N represents the number of generated molecules and $T(m_{1},m_{2})$ represents the Tanimoto similarity between molecule $m_{1}$ and molecule $m_{2}$. The greater, the better.

logP, the water-octanol partition coefficient, is a ratio of a chemical’s concentration in the octanol phase to its concentration in the aqueous phase of a two-phase octanol/water system. According to the Rule of 5(RO5) proposed by Lipinski (Lipinski et al. 2001), logP should be <5.

TPSA refers to the total surface area of all polar atoms. It measures the drug’s ability to permeate cell membranes. Molecules with a TPSA > 140 ${Å}^{2}$ have a limited ability to permeate cell membranes.

A detailed discussion of property-controllable generation can be found in Section 3.3.

## 3 Experiment

### 3.1 Dataset and parameter setting

We adopted the dataset popularly used in previous works (Luo et al. 2021). Built by Luo et al. (2021) based on binding pose RMSD (i.e. RMSD < 1 Å), it contains over 100 000 protein–ligand docking pairs, involving 2922 protein pockets and 13 839 ligand molecules. Each pair has a docking score measured by Autodock Vina (Trott and Olson 2010). Following the procedure proposed by Luo et al. (2021), we first clustered proteins at a sequence identity level of 30% by MMseqs2 (Steinegger and Söding 2017), such that two proteins coming from different clusters have ≤30% sequence identity (i.e. significantly different). Then, we took several clusters (i.e. 25 clusters) out of these clusters as the testing clusters and the remaining as the training clusters respectively. After that, we randomly extracted 100 000 protein–ligand pairs in the training clusters to build the model, where 99 000 pairs are labeled as the training pairs and 1000 pairs as the validation pairs. Last, we randomly selected 100 proteins (involving ∼18K protein–ligand pairs) from the testing clusters as the testing proteins (i.e. Reference) and assessed the performance of molecule generation w.r.t. proteins significantly different from the training proteins.

We used the training set to tune the learnable model parameters while determining the hyperparameters by empirical suggestions in other works.

Specifically, when constructing amino acid graphs in the protein embedding module, each node was initially represented as a 20-dimensional one-hot feature vector accounting for amino acid types, and the number of its nearest neighbors *k* = 30 as recommended by Ingraham et al. (2019). When encoding the edges between nodes, we used 64 Gaussian RBFs as suggested by Luo et al. (2021), where 64 centroids were taken at equal intervals between 0 and 25 Å and the width parameter of each RBF is the interval size (i.e. 25/64). Thus, each edge in amino acid graphs was represented as a 64-dimensional vector. Similarly, each node of the atom graph was initially represented as a 7-dimensional binary feature vector (Section 2.2) and the number of its nearest neighbors (*k*) was empirically assigned as 48. We used 64 Gaussian RBFs equidistantly spaced from 0 to 15 Å and set the width parameter to 15/64. As a result, each edge in atom graphs was also represented as a 64-dimensional vector. Finally, as Kreuzer et al. (2021) suggested, we collected the eigenvectors w.r.t. 8-smallest eigenvalues of the Laplace matrix as position codes.

In the dual-view encoder, each of the encoders contains 6 tandem encoding units, of which each unit is composed of 4 heads of attention layers. The hidden dimensions of both nodes and edges were set as 256. The dimensions of Query and Key in both the encoder and the cross-fusion module were set as 32, while the dimension of Value was set as 64. In addition, the feedforward network contains 1024 neurons.

In the molecule embedding module, each token (including tags and properties) in SMILES strings was initially represented as a 112-dimensional binary vector, including the beginning tag(1-d), the ending tag(1-d), the non-overlapping tokens w.r.t. SMILES strings (110-d) (Section 2.4). In the decoder, we set the length of token strings by the maximum length of SMILES sequences (i.e. 200). In addition, the parameters in the attention module in the decoder adopt the same values as those in the encoder.

When training our model, we set the batch size as 4, the initial learning rate α as 1e−4, and selected Adam as the optimizer. To accelerate the optimization, we adopted a decay strategy to regulate the learning rate as follows. If the loss of the validation set is not decreased within 5 iterations, $\alpha^{*}=0.6\alpha$ until it reaches 1e−5. We validated the model every 1000 training iterations and stopped the training if the loss does not decrease significantly within 20 validation iterations.

### 3.2 Method comparison

We assessed the performance of our CProMG by comparison with five state-of-the-art (SOTA) protein-oriented generative approaches, which including LiGANN (Skalic et al. 2019), 3D-SBDD (Luo et al. 2021), Pocket2Mol (Peng et al. 2022), naïve Transformer-based (Grechishnikova 2021), and AlphaDrug(BS) (Qian et al. 2022). These recently published approaches are briefly summarized as follows. LiGANN trained a GAN to generate 3D shapes of molecules, which match corresponding protein pocket shapes in topological complement, and then decoded the generated ligand shapes into multiple candidate SMILES strings by a captioning network. 3D-SBDD designed a 3D generative model to estimate the probability density of atom occurrences in the 3D binding space, and performed an auto-regressive sampling scheme on the binding spatial locations assigned with higher probabilities to generate 3D coordinates of molecules in a 3D grid atom by atom. Pocket2Mol designed an E(3) equivariant neural network to capture spatial and bonding relationships between atoms in the binding pocket and directly generated 3D coordinates of small molecules in continuous space. The naïve Transformer-based method directly applied the vanilla Transformer to generate novel molecule SMILES strings for specific amino acid sequences. Following this work, AlphaDrug improved the vanilla Transformer by skipping connections from its encoders to decoders. In addition, we employed DUD-E (dude.docking.org) to generate decoy molecules (denoted as Decoy) of the reference ligands which bind to the testing proteins.

Since those approaches adopt the same dataset, to make a fair comparison, we used the default values of parameters as those in the original papers in the comparison. For each protein in the independent testing set, top-10 molecules were generated for comparison.

It is the prime requirement that generated molecules bind to specific proteins with high affinities. Thus, we principally set the expected binding affinity as VS≤-7.5. Meanwhile, we expected two hard drug-like properties (i.e. QED≥0.6, SA≤4.0) to ensure that generated molecules are of high drug-likeness and easy to be synthesized respectively. A detailed investigation on controlling more properties can be found in Section 3.3. The generation performance was, on average, measured by the first five metrics, including VS, SA, HAR, QED, and Diversity. For both VS and SA, the less, the better. For the remaining, the greater, the better. In addition, we list the average results recorded (denoted as “Reference”) in the independent dataset as the baseline.

The results show that our CProMG significantly outperforms the Reference and other generative methods over all the metrics (Table 1, where *P*-values achieved by two-tailed *t*-tests are in parenthesis). In addition, since both HAR and Diversity are global metrics, the calculation of *P*-value is inappropriate for them. Especially, it reveals that our CProMG controlling VS, QED, and SA achieves the lowest VS, the lowest QED, and the highest SA as expected. In contrast, since these SOTA approaches cannot control the generation of molecules in terms of drug-like properties, they achieve sharply worse VS, QED, and SA. Therefore, the comparison demonstrates the superiority of our CProMG.

**Table 1.** Comparison with state-of-the-art approaches.

Method	VS	QED	SA	HAR (%)	Diversity
Reference	−7.550 (3.2e−2)	0.476 (7.7e−23)	3.453 (2.0e−5)	–	–
LiGANN	−6.144 (2.5e−269)	0.371 (0.0)	4.787 (1.9e−88)	23.8	0.655
3D-SBDD	−6.344 (1.2e−174)	0.502 (0.0)	3.912 (1.8e−39)	29.1	0.742
Pocket2Mol	−7.288 (2.9e−92)	0.563 (6.6e−233)	3.205 (1.4e−7)	54.2	0.688
Transformer	−7.385 (1.3e−33)	0.512 (1.6e−119)	2.756 (2.9e−11)	49.3	0.725
AlphaDrug	−7.393 (1.2e−29)	0.507 (1.1e−118)	**2.620** (3.1e−14)	50.1	0.727
Decoys	−6.737 (2.1e−15)	0.539 (3.5e−66)	3.830 (5.0e−69)	29.6	0.739
CProMG	**−7.644**	**0.741**	2.884	**55.5**	**0.757**

Note: Boldface values represent the best values of the metric.

### 3.3 Property-controllable generation

In this section, we investigate how well CProMG controls the molecule generation w.r.t. drug-like properties in a progressive manner including four scenarios. The first scenario, denoted as CProMG-w/oC, removes the controls of both binding affinity and properties. The second one, denoted as CProMG-V, keeps the control of binding affinity without property control by expecting VS≤-7.5. The third one, denoted as CProMG-VQS, sets the control of two hard properties QED and SA by expecting QED≥0.6 and SA≤4.0, based on binding affinity control. The last one, denoted as CProMG-VQSLT, sets an extra control of two soft properties LogP and TPSA by expecting $\mathrm{LogP}=\left\{2.0,\;4.0\right\}\;\mathrm{and}\;\mathrm{TPSA}=\left\{40.0,\;80.0\right\}$, based on the third scenario. Thus, the last strategy contains four settings.

**Figure 2.**
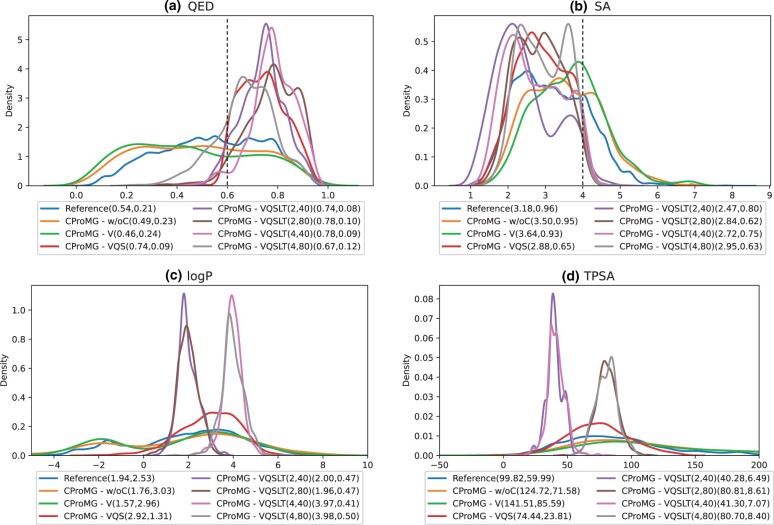
Property distributions of conditionally generated molecules. (a) QED, (b) SA, (c) logP, (d) TPSA. Both the reference distribution and the distributions of seven controlling scenarios are illustrated. Both the means and the standard deviations of distribution curves are annotated in parentheses following scenario names. Note that these values are annotated in the second parenthesis in the scenarios of CProMG-VQSLT since their first parenthesis annotates LogP values and TSPA values. In addition, the thresholds of QED and SA are marked in (a) and (b).

The overall results of the comparison are listed in Table 2 and its details are illustrated by the distributions of metric values in Fig. 2. The comparison reveals significant findings as follows.

**Table 2.** Comparison of property controlling strategies.

Strategy	VS	QED	SA	HAR	Diversity
Reference	−7.550	0.476	3.453	–	–
CProMG-w/oC	−7.384	0.488	3.500	48.8	0.736
CProMG-V	−7.849	0.452	3.655	56.8	0.721
CProMG-VQS	−7.644	0.741	2.884	55.5	**0.757**
CProMG-VQSLT(2,40)	−6.587	0.741	**2.467**	29.2	0.736
CProMG-VQSLT(2,80)	−7.562	0.776	2.836	51.0	0.745
CProMG-VQSLT(4,40)	−7.717	**0.782**	2.722	53.3	0.721
CProMG-VQSLT(4,80)	−**7.977**	0.673	2.950	**61.3**	0.730

*Note*: CProMG-VQSLT(*, #) accounts for a specific LogP value(*) and a specific TSPA value (#). Boldface values represent the best values of the metric.

1. Even without property control, CProMG can generate molecules, which have approximate properties to those of reference molecules. For example, their QED/SA distribution (orange/green curves in Fig. 2) is similar to that of reference molecules (blue curves).
2. In contrast, CProMG with property control can generate molecules having better properties. Specifically, the controls of binding affinity, QED, and SA always contribute to high-binding affinities, high QEDs, and low SAs as expected (Table 2, Fig. 2a and b). For example, ∼99.18% of the novel molecules generated by CProMG-VQS shows QED≥0.6, while ∼99.19% shows SA≤4.0. Moreover, the controls of LogP and TPSA make generated molecules own the right values of LogP and TPSA around their expectations (Fig. 2c and d). For example, all cases of CProMG-VQSLT show the peaks of value distributions at the expected property values with small dispersions.
3. It exists a trade-off among the controls over diverse properties. Table 2 exhibits that the smaller LogP results in a smaller SA (better), a smaller QED (worse), and a bigger VS (worse), while the greater TPSA causes a smaller VS (better) and a bigger SA (worse).
4. As shown in Table 2, neither the binding-affinity control nor drug-like property control increases the Diversity, which depends on other modules of CProMG.

### 3.4 Ablation studies

In this section, we investigated how well each component of our model contributes to the prediction by ablation studies in the case of controlling the binding affinity. We made four variants of our original model by only considering the control of binding affinity since it is the prime requirement. Each variant masks one block of CProMG, which helps generate molecules having high-binding affinity with specific proteins. The first one only considers the atom view, ignoring the amino acid view (denoted as w/o AA), while the second ignores the atom view (denoted as w/o Atom). The third removes the Laplacian position encoding (denoted as w/o LPE). The last (denoted as w/o C) removes the conditional control of binding affinity (i.e. docking score).

The comparison shows that CProMG significantly outperforms all the variants on the VS and HAR (Table 3, where *P*-values are in parenthesis). The results demonstrate that both the amino acid view and the atom view contribute to protein-oriented molecule generation because they provide coarse-grained representations and fine-grained representations of protein binding pockets respectively. Also, the Laplacian positional encoding has a untrivial contribution to protein-oriented molecule generation because it can extract the unique positional representations of protein binding pockets. Last, the results reveal again that the conditionally control of binding affinity is crucial to generating molecules with high-binding affinity to specific proteins.

**Table 3.** Ablation comparison.

	Ref.	CProMG-V	w/o AA	w/o Atom	w/o LPE	w/o C
VS	−7.550 (3.6e−2)	**−7.849**	−7.593 (1.2e−5)	−7.441 (4.3e−9)	−7.729 (4.3e−2)	−7.384 (2.6e−11)
HAR (%)	–	**56.8**	50.6	49.7	53.2	48.8

Note: Boldface values represent the best values of the metric.

In general, the amino acid view encoder, the atom view encoder, the Laplacian position encoding and the property control play indispensable roles when generating protein-oriented molecules with desired binding affinity. Similarly, we also investigated how the conditional control of other properties affects the molecule generation. Similar results were found.

### 3.5 Case studies

**Figure 3.**
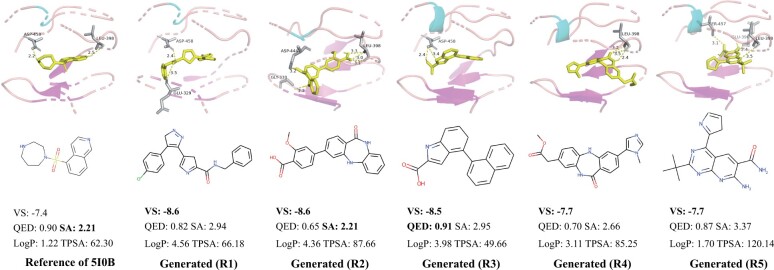
Case study. The reference molecule is located in the left column while top-5 generated molecules are listed in a descending ordered w.r.t. VS in the remaining columns. Both their binding affinity (VS) and four drug-like properties (QED, SA, LogP, and TPSA) are annotated as well.

As Peng et al. (2022) did, we selected the protein (PID: 5I0B) in the testing set as a case study. Its mutations are detected in multiple tumor issues. After running CProMG-VQS, we selected its top-5 generated molecules in terms of VS, and apply RDKit to calculate their values of QED, SA, LogP, and TPSA (Fig. 3). We found that their SA = 2.827 and QED = 0.789 on average. In addition, each molecule satisfies the conditions of QED≥0.6;SA≤4.0, while its LogP and TPSA fits the RO5. This demonstrates that the generated compounds are easy to be synthesized and have good drug-like properties.

Looking into the binding pocket by the Autodock Vina (Trott and Olson 2010). We found that the reference inhibitor molecule has stable polar contacts with the two surrounding residues (i.e. ASP-458 and LEU-398). Due to the dual-view fusion encoder and the decoder, five generate molecules retain polar contacts with at least one of these residues. Moreover, there are also polar contacts with other surrounding residues, such as GLU-323 in R1, GLY-330 in R2, and both SER-457 and GLU 396 in R5.

In addition, the structures of the generated molecules are significantly dissimilar to that of the reference molecule (i.e. 0.221, 0.222, 0.245, 0.228, and 0.263 in terms of Tanimoto similarity). The results validate that the molecules generated are novel.

In summary, the case study demonstrates that novel molecules generated by our CProMG can not only bind to given specific proteins in high affinity but also own desired drug-like properties.

## 4 Conclusion

In this article, we have proposed a protein-oriented generative framework for molecule generation (CProMG) under the control of high-binding affinity and desired drug-like properties. CProMG contains a 3D protein embedding module, a dual-view protein encoder, a molecule embedding module, and a novel drug-like molecule decoder. This end-to-end framework can address two existing issues, including inadequate protein representation and incontrollable generation in properties.

The comparison with recently published deep generative methods demonstrates the superiority of CProMG. Moreover, the progressive Property-control, the ablation studies as well as the case study validate its contributions. First, CProMG provides a comprehensive framework to generate novel molecules for given proteins with high-binding affinities and desired drug-like properties. Secondly, by fusing the hierarchical views of proteins, it significantly enhances the characterization of protein binding pockets by associating amino acid residues with their comprising atoms. Thirdly, the protein-interactive multi-head attention block in the decoder calculates the proximity of molecule tokens to protein residues and atoms, such that crucial interactions between protein pockets and molecules can be captured.

In summary, we believe that our study provides new insights into molecule generation for *de novo* drug design.
